# Immune Phenomena in Myeloid Neoplasms: An “*Egg or Chicken”* Question

**DOI:** 10.3389/fimmu.2021.751630

**Published:** 2021-09-29

**Authors:** Wilma Barcellini, Bruno Fattizzo

**Affiliations:** ^1^ Hematology Unit, Fondazione IRCCS Ca’ Granda Ospedale Maggiore Policlinico, Milan, Italy; ^2^ Department of Oncology and Hemato-Oncology, University of Milan, Milan, Italy

**Keywords:** myelodysplastic syndromes, acute myeloid leukemia, myeloproliferative neoplasms, microbiome, autoimmunity, immunodeficiencies

## Abstract

Immune phenomena are increasingly reported in myeloid neoplasms, and include autoimmune cytopenias/diseases and immunodeficiency, either preceding or complicating acute myeloid leukemia, myelodysplastic syndromes (MDS), chronic myeloproliferative neoplasms, and bone marrow failure (BMF) syndromes. Autoimmunity and immunodeficiency are the two faces of a dysregulated immune tolerance and surveillance and may result, along with contributing environmental and genetic factors, in an increased incidence of both tumors and infections. The latter may fuel both autoimmunity and immune activation, triggering a vicious circle among infections, tumors and autoimmune phenomena. Additionally, alterations of the microbiota and of mesenchymal stem cells (MSCs) pinpoint to the importance of a permissive or hostile microenvironment for tumor growth. Finally, several therapies of myeloid neoplasms are aimed at increasing host immunity against the tumor, but at the price of increased autoimmune phenomena. In this review we will examine the epidemiological association of myeloid neoplasms with autoimmune diseases and immunodeficiencies, and the pivotal role of autoimmunity in the pathogenesis of MDS and BMF syndromes, including the paroxysmal nocturnal hemoglobinuria conundrum. Furthermore, we will briefly examine autoimmune complications following therapy of myeloid neoplasms, as well as the role of MSCs and microbiota in these settings.

## Introduction

**Figure 1:**
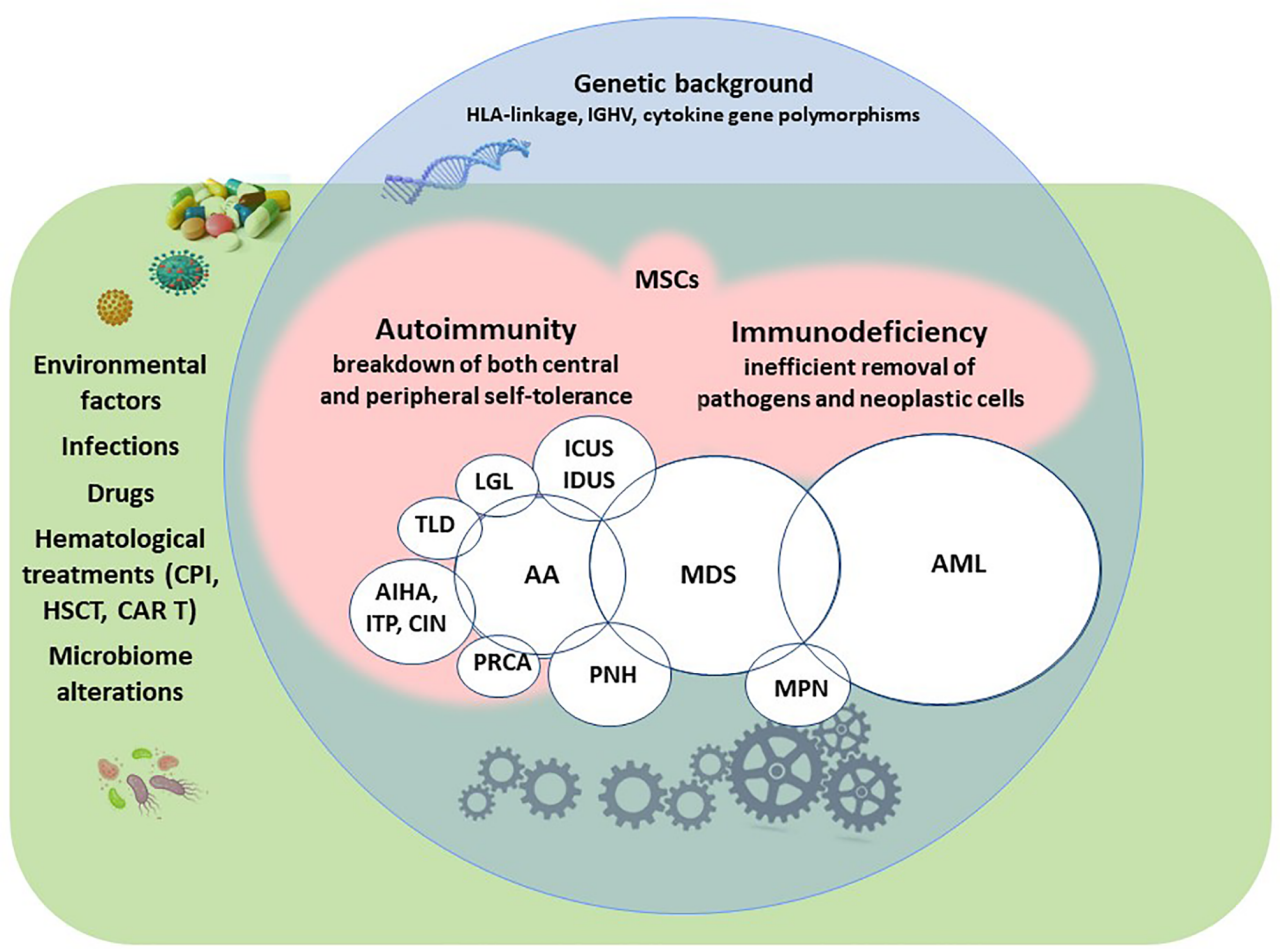
Autoimmunity and immunodeficiency in myeloid neoplasms and associated conditions. AML, acute myeloid leukemia; MDS, myelodysplastic syndromes; MPN, myeloproliferative neoplasms; AA, aplastic anemia; ICUS, idiopathic cytopenia of undetermined significance; IDUS, idiopathic dysplasia of undetermined significance; PNH, paroxysmal nocturnal hemoglobinuria; PRCA, pure red cell aplasia; AIHA, autoimmune hemolytic anemia; ITP, immune thrombocytopenia, CIN, chronic idiopathic neutropenia; TLD, telomeres diseases; LGL, large granular lymphocyte lymphoproliferative syndromes; CPI, checkpoint inhibitors; HSCT, hematopoietic stem cell transplantation; CAR T, chimeric antigen receptor T-cells; MSCs, mesenchymal stem cells.

The immune system is broadly involved in maintaining homeostasis, either by fighting infectious agents or controlling tumor growth. Autoimmunity and immunodeficiency are the two faces of a dysregulated immune tolerance and surveillance and may result, along with contributing environmental and genetic factors, in an increased incidence of tumors (1). Autoimmunity is primarily the consequence of an improper self-directed immune reaction, whilst immunodeficiency is the inability to efficiently eliminate infectious pathogens or neoplastic cells, and both may result in severe and life-threatening diseases. There is a delicate balance between immune-defense mechanisms and autoimmune reactivity, as recently highlighted by the autoinflammatory response and autoimmune complications following therapy with checkpoint inhibitors (CPI) and chimeric antigen receptor (CAR) T-cells (2). The association between lymphoproliferative disorders and peripheral immune-mediated cytopenias is well known, along with the underlying pathogenic mechanisms (3). The presence of autoimmune phenomena/diseases is less investigated in myeloid neoplasms, although reported in bone marrow failure (BMF) and myelodysplastic syndromes (MDS), as well as in chronic and acute myeloproliferative diseases (2). Of note, autoimmune phenomena may be a spurious serologic finding without clinical consequences, or even represent a favorable response aimed at eliminating damaged/harmful self-structures (1). On the other hand, the association of immunodeficiency with tumors is well known, together with the role of consequent chronic/relapsing infections that may fuel both autoimmunity and immune activation. The latter may be exaggerated, ineffective and potentially harmful, triggering a vicious circle among infections, tumors and autoimmune phenomena. More generally, there is increasing interest on the role of the immune system in the generation of a permissive or hostile microenvironment for tumor growth, which has recently involved also the microbioma and the mesenchymal stem cells (MSCs) (4–6). This review will examine immune phenomena (autoimmunity and immunodeficiency) in myeloid neoplasms, including MDS, BMF syndromes, acute myeloid leukemia (AML), and chronic myeloproliferative neoplasms (MPN). We will also focus on the several overlapping conditions, highlighting the continuous and mutual cross-talk between the immune effectors and the neoplastic cells (**Figure 1**).

## Epidemiological Association of Autoimmune Diseases and Myeloid Neoplasms

Several autoimmune diseases (AID) and, less frequently, autoimmune cytopenias (AIC) have been described in myeloid neoplasms. These include systemic and organ specific disorders, such as rheumatoid arthritis (RA), systemic lupus erythematosus (SLE), vasculitis, thyroid autoimmune diseases, Sjogren syndrome (SS), autoimmune hemolytic anemia (AIHA), immune thrombocytopenia (ITP), pure red cella aplasia (PRCA), and immune-mediated hemostatic disorders (2, 7–10). The diagnosis may be challenging due to the great clinical heterogeneity and variable organ involvement of AID, whose signs/symptoms may be confounded with those of the hematologic malignancy. Likewise, diagnosis of AIC may be complicated by overlapping conditions like chemotherapy, bone marrow infiltration, and transfusion support (2). Additionally, straightforward diagnostic tests are lacking, particularly for AID, and several diagnostic pitfalls exist for AIC as well. Among myeloid neoplasms, MDS and chronic myelomonocytic leukemia (CMML) are complicated in up to 20-30% by vasculitis subtypes, more commonly Behçet’s-like syndrome, relapsing polychondritis, polyarteritis nodosa and giant-cell arteritis (2, 9). CMML is also frequently complicated by ITP either concomitant or preceding its diagnosis, while AIHA and PRCA are occasionally observed (9). Regarding MPN, including myelofibrosis (MF), various cases of RA, dermatomyositis, polyarteritis nodosa, multiple sclerosis, inflammatory bowel disease, and primary biliary cirrhosis have been reported (11, 12). AML is also occasionally associated with AID as well as with AIC (13, 14). Finally, case-reports/small series of immune-mediated hemostatic disorders have been described in MDS, CMML, MPN, and AML. These included acquired hemophilia A, thrombotic thrombocytopenic purpure, and anti-phospholipid syndrome, that may be life-threatening (2). On the other hand, there is evidence that patients with prior systemic autoimmune rheumatic diseases have an increased risk for the development of hematological malignancies, particularly lymphomas and MDS. This has been reported for RA, SS, SLE, ITP, myasthenia gravis, and giant cell arteritis, suggesting that the immune dysregulation underlying the autoimmune disease may be involved in the generation of a “tumor permissive” soil, although the contribution of treatment with immunosuppressive/cytotoxic drugs cannot be excluded (15–17).

## Immunodeficiency and Myeloid Neoplasms

Immunodeficiency is a broad concept that may involve the deficiency of one of the several arms of the immune system (1). Primary immunodeficiency syndromes (PID) represent a complex and heterogeneous category comprising more than three-hundred distinct disorders, mostly congenital, increasingly diagnosed through genetic and immunologic tests. They are grouped according to the predominant deficiency, including T-B severe combined defects, antibody, complement, neutrophils, and cytokine deficiencies, and also encompass hematologic conditions such as Fanconi Anemia, Diamond-Blackfan anemia, Familial Hemophagocytic Lymphohistiocytosis, and Wiskott-Aldrich syndrome (immunodeficiency with congenital thrombocytopenia). Other immunodeficiencies are associated with somatic mutations, such as the autoimmune lymphoproliferative syndrome and the RAS-associated autoimmune leukoproliferative disease (18–20). An increased risk of developing acute leukemias (mostly T-cell derived), lymphomas and MDS, as well as other solid cancers have been described in most of the PID listed above, underlying the concept that an efficient immunosurveillance is pivotal in preventing tumorigenesis. Moreover, continuous/relapsing infections consequent to immunodeficiency may sustain a state of chronic hyper-inflammation, which in turn may favor tumor growth (18–20). An interesting player in controlling neoplastic expansion is the complement system that may be exploited by monoclonal antibodies directed against tumor antigens, increasingly used as therapeutic tools in various cancers (21). An example of interplay among immunodeficiency, autoimmunity and cancer is the Kabuki syndrome, a rare genetic disorder with specific facial features, intellectual disability, and increased frequency of infections, autoimmune diseases and neoplasias. The syndrome is caused by mutations in the KDM6A or KMT2D genes, which are involved in the early differentiation of mesenchymal cell lineage and in the development of tolerance and immune system maturation (22, 23). Another example is the MIRAGE syndrome (Myelodysplasia, Infection, Restriction of growth, Adrenal hypoplasia, Genital problems, and Enteropathy) which is caused by mutation in the SAMD9 gene, a regulator of inflammatory response acting as a downstream target of TNF-alpha signaling (24). Finally, the group of telomeropathies comprises several heterogeneous defects in the telomere maintenance machinery, characterized by a variable clinical phenotype and genetic penetrance, and by great susceptibility to environmental factors. Among the several, Dyskeratosis Congenita, idiopathic pulmonary fibrosis, and familial liver cirrhosis, show variable overlap with autoimmune diseases and BMF syndromes, as well as increased risk of infections and hematologic neoplasms (25, 26).

## Autoimmunity in BMF Syndromes: MDS, Hypoplastic MDS, and AA

Several lines of evidence support the relationship between BMF/MDS and autoimmunity, i.e. their epidemiologic association, the response to similar immunosuppressive therapies, and the existence of common immune-mediated physiopathologic mechanisms (27–29). The latter include bone marrow suppression by T-cells, cytokine dysregulation, and apoptosis of hematopoietic precursors. Aplastic anemia is the prototype of an immune-mediated attack against BM, with several effectors involved, such as activated cytotoxic T cells, increased production of type 1 cytokines, reduced T reg, and enhanced apoptosis *via* Fas/FasL. Moreover, defects of the innate immunity and of the hematopoietic niche are also reported as important pathogenic mechanisms. The most relevant evidence supporting the autoimmune pathophysiology is the response to immunosuppressive therapy (IST) and the requirement, in most cases, of continuous immunesuppression to maintain response (30). At the boundaries of the classic BMF syndromes, and with frequent overlap, there are other diseases in which several immunologic abnormalities are increasingly reported. These include pure red cell aplasia, pure white cell aplasia, amegakaryocytic thrombocytopenia, and the telomere diseases, all definitely cytopenic; however, other diseases, such as large granular lymphocyte lymphoproliferative (LGL) syndromes, and hemophagocytic lymphohistiocytosis (HLH), may manifest with autoimmune cytopenias along with proliferative features (31–35). Additionally, the landscape of BMF syndromes has been enriched with the recently described idiopathic cytopenia/dysplasia of undetermined significance (ICUS/IDUS) and the hypoplastic MDS, which again share common immune-mediated pathogenic mechanisms and a cytopenic phenotype (27, 32, 36, 37).

There is growing interest about the presence of somatic mutations in autoimmune/autoinflammatory conditions. It is known that mutational burden increases with age, as described in the so called clonal hematopoiesis of indeterminate potential (CHIP) or clonal cytopenia of undetermined significante (CCUS). Recently, DNMT3A, TET2 and ASXL1 mutations were found in 29.5%, 15.0% and 3.5% of studied patients, with a striking association with autoimmune diseases (38). Regarding BMF, a variable combination of somatic mutations has been largely described: in MDS the most frequently observed involve the splicing genes SF3B1, SRSF2, U2AF1, ZRSR2, the DNA methylation genes DNMT3A, TET2, IDH1, IDH2, and the chromatin modification genes ASXL1, EZH2, KDM6A (39). In AA the genomic landscape is more shaded, with less MDS-associated and more frequent typical paroxysmal nocturnal hemoglobinuria (PNH)-related PIGA mutations (40, 41), while in hypoplastic MDS the picture is somehow in between MDS and AA (32). More recently, mutations have been detected even in lymphoid cells of BMF patients. For instance, JAK-STAT and MAPK pathways mutations have been described by single-cell sequencing in CD8+ T cells of AA patients, and the mutational burden was associated with CD8+ T-cell clonality (42). It may be speculated that mutations accumulate in the pathogenic immune effectors, which are the most activated and replicating cell types. Finally, STAT3 somatic mutations have been reported in CD8+ T cells in LGL, and in other autoimmune “benign” conditions, such as rheumatoid arthritis/Felty’s syndrome, multiple sclerosis, and celiac disease (43).

## The PNH Conundrum

One of the key mechanisms of PNH pathogenesis is the escape of GPI-negative hematopoietic precursors from a GPI-directed autoimmune attack. This hypothesis is supported by the known association of PNH and AA, and by the reported increase of the PNH clone after IST in AA (44, 45). However, inactivating mutations of PIG-A do not “per se” confer a selective growth advantage to hematopoietic precursors, since they are found in several conditions including healthy subjects, without causing overt disease. Thus, further events (additional cooperating mutations)? are thought to be necessary for the expansion of the PIG-A mutant clone. Additionally, the bone marrow environment (dominated by an auto-immune signature) seems to play an important role in a further growth advantage of the PNH clone (46). Bone marrow microenvironment and autoimmune phenomena may play a role also in MDS, where mainly small PNH clones are described, without an overt hemolytic disease (45). Consistently, anti- erythroblast antibodies have been demonstrated in about 2/3 of early MDS together with increased values of the pro-apoptotic protein Bax and decreased levels of Bcl‐2 levels, and their BM culture supernatants induced dyserythropoietic signs, erythroblastic clustering, and increased overall in cultured normal BM (47, 48). Furthermore, small PNH clones have been demonstrated in hypomegakaryocytic thrombocytopenia (49, 50) and chronic idiopathic neutropenia (51), two conditions hardly distinguishable from ICUS and with autoimmune reactivity against BM precursors. Finally, small PNH clones have been reported also in a considerable proportion of AIHAs, conferring a prominent hemolytic pattern and a higher thrombotic risk to the disease. The presence of a PNH clone was also associated with a different cytokine signature (reduced levels of IFN-γ and IL-17) as compared to PNH-negative AIHA cases (52). In AIHA the clinical picture is dominated by an immune attack directed against peripheral erythrocytes; however, in cases with reticulocytopenia and severe onset the immune attack is also directed against bone marrow precursors. Additionally, chronic/relapsing AIHA show a possible evolution to ICUS, IDUS or BMF syndromes over time, suggesting a shift from “peripheral” to “central” autoimmunity (27, 28, 53, 54). Altogether these findings support the idea that the PNH clone may be the “immunological scar” of an immune attack directed against BM precursors.

## Autoimmune Complications Following Therapy of Myeloid Neoplasms

Historically IFN-alpha has been used in MPN, including chronic myeloid leukemia (CML), systemic mast cell disease, and hypereosinophilic syndrome, and has been associated with the occurrence of several AID and AIC. Several autoimmune side effects have been described, ranging from spurious autoantibody formation to overt diseases, such as AIHA, ITP, thrombotic thrombocytopenic purpura, hypo- or hyper thyroid disorders, SLE, RA, and Behçet’s disease (55). Tyrosine kinase inhibitors (TKIs) have certainly changed the therapeutic approach to MPN, and their risk-benefit balance is well established, with mainly infectious concerns (56). However, most of their effects on the complex regulations of the immune system are far from being fully elucidated. They have immunosuppressive effects on monocyte/macrophage functions, dendritic cell maturation, and lymphocyte subsets, but also exert an immunomodulatory activity inducing monocyte type 1 polarization, increased NK function and T-lymphocyte activation. This has provided a rationale for investigating a possible therapeutic use in autoimmune diseases (57). On the other hand, imatinib has been associated with the occurrence of aplastic anemia, and nilotinib and dasatinib implicated in the development of immune-mediated liver injury, SLE, panniculitis, and neurologic demyelinating disease. The harmful effects of potent immunomodulation are even more marked with aggressive therapies such as BM transplantation, where several autoimmune complications are thought to be the consequence of the “immunological storm” elicited by the battle against tumor cells (58–60). The complexity of the immunological perturbation following allogenic BM transplantation is maximally reflected in the well known graft-versus-host-disease, which resembles progressive systemic sclerosis and vasculitis of the skin, gastrointestinal tract, liver, lungs, and kidneys. Additionally, CAR T-cells, although mainly studied in lymphoid conditions, are recently considered also for acute myeloid leukemia (61). Well known toxicities of CAR T-cells also derive from a complex immune dysregulation and include the cytokine release syndrome, the immune effector cell-associated neurotoxicity syndrome, and the hemophagocytic lymphohistiocytosis; later toxicities comprise prolonged cytopenias and hypogammaglobulinemia (62). Finally, the difficulty of a balanced immune stimulation is also highlighted by the autoimmune complications following therapy with check point inhibitors in solid and hematologic tumors (63, 64).

## Mesenchymal Stem Cells in Autoimmune Diseases and Myeloid Neoplasms

Mesenchymal stem cells are key constituents of the BM niche able to differentiate in various tissues and to exert several immunomodulatory activities (5, 6). They appear to play a pivotal role against tumors and infections through a variety of properties including anti-inflammatory, regenerative, angiogenic, anti-fibrotic, anti-apoptotic, and anti-oxidative stress activities. Conversely, there is growing evidence that MSCs from patients with myeloid neoplasms may differently support leukemic growth and protect the leukemic cell from apoptosis or chemotherapy-induced cell death. MSCs behavior may be due to the bidirectional crosstalk between the leukemic cell and the BM niche, that realizes through several cytokines, chemokines and other soluble factors (CXCR2, CXCR4, IL6R, LFA, VLA4, RANK and FAT/CD36) as described in AML (65). On the other hand, MSCs may harbor intrinsic alterations, as recently reported for MDS and AA. These included dysregulated proliferation/apoptosis (prevalent in MDS), decreased angiogenesis (prevalent in AA), and immunosuppressive functions, with a shift from type 1 (pro-inflammatory) to type 2 (anti-inflammatory/tumor-educated) MSC profile (5). Finally, MSCs may be involved in resistance even to novel treatments, as observed in CML. In this setting, MSCs favor the immune escape of residual leukemic cells causing resistance to TKIs, that may overcome by interferon-α (66). On the whole, the immunomodulatory properties of MSCs and their possible allogeneic/unmatched use have promoted several clinical trials in various autoimmune disorders, including aplastic anemia, Crohn’s disease, multiple sclerosis, inflammatory liver and pulmonary diseases, neurodegenerative disorders, as well as in graft rejection and graft-vs-host-disease (67). Limitations of MSCs as cell therapy include handling difficulties, safety issues, and high economic cost. Thus, the use of MSC-derived secretome products is increasingly pursued, although the choice of the ideal MSC type and the standardization of production strategies need to be defined.

## Microbiome in Autoimmune Diseases and Myeloid Neoplasms

There is growing evidence on the role of microbiome in regulating several homeostatic processes, such as metabolic pathways, synthesis of vitamins and fat storage, as well as self-tolerance, immune surveillance for tumors, and host defense against pathogens. Alterations of the microbiome have been associated with the development of autoimmune diseases, neoplasms, and infections and their treatments (68). Beyond data on BM transplantation, it has been shown that a permissive microbiota, i.e. the microorganisms that colonize various districts of the body, is associated with acute leukemias, lymphoma, and multiple myeloma (4). Of note, microbiome changes may be induced by the leukemic cell itself, by chemotherapy or antibiotics, or by superimposed infections, adding further complexity to the topic. Alterations of the microbiome have been reported also in autoimmune cytopenias and aplastic anemia (69–71). Helicobacter pylori colonization has long been associated with ITP, although it is not strictly considered a microbiome alteration (4). More recently, in ITP and chronic idiopathic neutropenia, a peculiar microbiota composition has been identified, possibly predictive of response to therapy (72). Similarly, alterations of the microbiota have been reported in SLE, RA, Multiple Sclerosis and Type-1 diabetes (73). Moreover, Parvovirus B19, hepatitis viruses, CMV and EBV have been associated with transitory forms of AA, probably due to a molecular mimicry between foreign and self-antigens or polyclonal immune stimulation. In addition, the presence of chronic inflammation, the alteration of epithelial barriers, and the dissequestration of self-antigens driven by an altered microbioma are also thought to contribute to the development of autoimmune diseases and neoplasms (4). In the next future the availability of NGS techniques will improve the ability to analyze the microorganisms, providing new insights in this fascinating area, and improving the knowledge of disease pathogenesis, complications, and therapy outcome.

## Autoimmune Phenomena

By definition autoantibodies are antibodies that react with self-antigens (1), although not invariably associated with AID and AIC. It is largely known that low-affinity IgM and, occasionally, low titer IgG autoantibodies are detected in healthy individuals. They comprise rheumatoid factors, antinuclear-, and even anti-RBC and anti-platelets antibodies, without a clinically overt disease. Additionally, natural autoantibodies, mainly polyreactive IgM with a moderate affinity for self-antigens, may provide a first line defense against infections and have housekeeping functions by recognizing apoptotic cells and promoting their phagocytic clearance (74). This phenomenon has been extensively studied in thalassemias and congenital hemolytic anemias, hypothesizing a physiologic role in the clearance of debris of lysed cells (75). In this view natural autoantibodies may concur to the opsonization and removal of potentially harmful elements, including tumor cells. The role of natural autoantibodies is not known in hematologic neoplasms, with the isolated exception of chronic lymphocytic leukemia (CLL). Autoimmunity and immunodeficiency are a hallmark of CLL, with a relentless accumulation of anergic, self-reactive CD5+ B cells, which produce several polyreactive natural autoantibodies (76). As regards myeloid neoplasms, it may be hypothesized that natural autoantibodies may contribute (or not) to the first-line defense of innate immunity that has been found deficient in most of these disease (77–79).

## Conclusion

Immune phenomena, both autoimmunity and immunodeficiency, are definitely an egg and chicken question in cancer, including myeloid neoplasms. The complexity of the issue is further increased by the heterogeneity of myeloid disorders that encompass truly malignant cells (as in acute leukemias) and more subtle diseases (as MDS and BMF syndromes). Likewise, immune-mediated phenomena are broadly represented, including immune-mediated cytopenias and hemostatic disorders, classic autoimmune diseases, and immunodeficiencies, as summarized in **Table 1**. The immune system is undoubtedly pivotal in preventing and controlling tumor growth and in direct killing of neoplastic cells. These functions are characterized by pleiotropism (several different activities are performed by a single effector depending on the setting) and redundancy (the same result is accomplished by different effectors). In this complex scenario, two main tasks are required to maintain homeostasis: tolerance versus self and surveillance against potential harmful noxae, i.e., infectious agents and tumors. However, microorganisms are not always dangerous and autoimmunity may be potentially useful in removing damaged/badly functioning cells. Autoimmunity occurs in peripheral blood and, less investigated, in bone marrow and lymphoid organs, and may be fundamental in maintaining homeostasis, provided its tight control and absence of over activation. Stretching the concept, the removal of a neoplastic cell may be considered a self-directed “autoimmune” reaction. Additionally, autoimmune phenomena, although not directly causative, may be deeply involved in the pathogenesis of myeloid neoplasms, particularly MDS and BMF. At variance, in AML, autoimmunity takes a second place in pathogenesis, whilst it can be clinically harmful. Finally, there are escape phenomena, like PNH clones, which can guarantee hematopoiesis, albeit deficient. More than 30 years ago a visionary scientist, J. Edwin Blalock, pioneer of neuroimmunoendocrinology, defined the immune system as our “circulating brain” (80), and this notion is still up to date and far away from being fully understood.

**Table 1:** Main evidences of immune system involvement in myeloid neoplasms.

Epidemiological associations with autoimmunity	- MDS is associated with systemic and organ specific disorders, such as RA, SLE, vasculitis, thyroid autoimmune diseases, SS, AIHA, ITP, PRCA, and immune-mediated hemostatic disorders in about 20% of cases. - CMML are complicated in up to 30% by vasculitis and ITP. - MPN and AML are occasionally complicated by autoimmune cytopenias and diseases.
Epidemiological associations with immunodeficiency	- AML and MDS are observed in patients with primary immunodeficiencies, including T-B severe combined defects, antibody, complement, neutrophils, and cytokine deficiencies.
Autoimmunity in BMF syndromes	- MDS, particularly low-grade hypoplastic type, is marked by autoimmune phenomena promoting apoptosis of hematopoietic precursors. - AA, which may evolve to MDS/AML, has a well-defined autoimmune pathogenesis against BM precursors (cytotoxic T cells, increased production of type 1 cytokines, and reduced T reg). - MDS and AA share common somatic mutations (mainly splicing genes, DNA methylation genes, and chromatin modification genes), although with different frequencies.
Overlapping syndromes	- MDS presenting with cytopenia and autoimmunity displays overlapping features with ICUS/IDUS, PRCA, white cell aplasia, amegakaryocytic thrombocytopenia, telomere diseases, LGL, and HLH.
PNH clones	- MDS, MPN and AML may display PNH clones, usually small or very small. - AA and peripheral autoimmune cytopenias are also associated with PNH clones.
Autoimmune complications following therapy	- MPN, particularly CML, has been historically treated with IFN-alpha, which induced several autoimmune complications (AIHA, ITP, TTP, and other autoimmune diseases). - MPN treated with TKIs (imatinib, nilotinib and dasatinib) may be complicated by immune-mediated disorders (AA, SLE, liver injury, and neurologic demyelinating disease). - AML and MDS subjected to more aggressive therapies (HSCT, CAR T, and CPI) may be complicated by several autoimmune cytopenias and other immune-mediated disorders (cytokine release, neurotoxicity syndrome, HLH).
Mesenchymal stem cells	- AML, MDS, and MPN show alterations of MSCs that may be implied in disease pathogenesis and resistance to therapy.
Microbiome	- AML is associated with alterations of the microbiome, either *per se* or because of chemotherapy, antibiotics and HSCT. Alterations of the microbiome have been reported in various autoimmune diseases.

AML, acute myeloid leukemia; MDS, myelodysplastic syndromes; MPN, myeloproliferative neoplasms; CMML, chronic myelomonocytic leukemia; AA, aplastic anemia; ICUS, idiopathic cytopenia of undetermined significance; IDUS, idiopathic dysplasia of undetermined significance; PNH, paroxysmal nocturnal hemoglobinuria; PRCA, pure red cell aplasia; LGL, large granular lymphocyte lymphoproliferative syndromes; HLH, hemophagocytic lymphohistiocytosis; AIHA, autoimmune hemolytic anemia; ITP, immune thrombocytopenia, TTP, thrombotic thrombocytopenic purpura; RA, rheumatoid arthritis; SLE, systemic lupus erythematosus, SS, Sjogren syndrome; IFN, interferon; TKIs, tyrosine kinase inhibitors; CPI, checkpoint inhibitors; HSCT, hematopoietic stem cell transplantation; CAR T, chimeric antigen receptor T-cells; BM, bone marrow; MSCs, mesenchymal stem cells.

## Author Contributions 

All authors listed have made a substantial, direct, and intellectual contribution to the work, and approved it for publication.

## Conflict of Interest

The authors declare that the research was conducted in the absence of any commercial or financial relationships that could be construed as a potential conflict of interest.

## Publisher’s Note

All claims expressed in this article are solely those of the authors and do not necessarily represent those of their affiliated organizations, or those of the publisher, the editors and the reviewers. Any product that may be evaluated in this article, or claim that may be made by its manufacturer, is not guaranteed or endorsed by the publisher.
